# 

**Published:** 1888-10

**Authors:** 


### BOOK NOTICES.

THE BANJO.—A dissertation by S. S. Stewart. The author of this little work, the first of the kind that has appeared, rates the banjo among the first of musical instruments. He also gives the particulars of a *banjo cure*, which certainly is an improvement on the faith or prayer cure, but what the "appropriate jestures" had to do in restoring the sleeping patient, it is difficult to conceive. Not being able to find the portrait of Miss Secor among the other notables we presume it occupies the place of honor on the title page. The author is a manufacturer of Banjos, Guitars, etc., and publishes the Banjo and Guitar Journal, 223 Church Street, Philadelphia.

We should like to see one of those Guitar's in our offiee.

THE SATELLITE of the ANNUAL OF THE UNIVERSAL MEDICAL SCIENCE.—This is a quarterly review of "the most important articles appearing in the Medical Press at large," which has now reached its second issue, and judging from the various subjects presented, includineg Medicine, Surgery and Operative Dentistry, the publication will be found of great value to each of these professions.

Philadelphia: F. A. DAVIS, Publisher, 1231 Filbert Street.

THE CENTURY MAGAZINE comes to hand with no signs of deterioration in its contributed articles or editorial matter. The Siberian papers grow in interest and Lincoln and his times expanding into a complete military history of the late Civil War.

The October issue contains a number of new and interesting articles including "Frontier Types," "Army Hospitals and Cases," with excellent illustrations, as usual.

CENTURY COMPANY, Union Square, New York City.

THE PEOPLE'S SAFE GUARD—is a monthly newspaper full of instructive reading. Published at 50 cents a year, by THE COM. PRINTING AND PUB. CO., Chicago, Ill.

BABY LAND AND OUR LITTLE MEN AND WOMEN.—D. LATHROP CO., Boston, 50c. per year. These admirable publications are a delight to our little folks, who cannot fail to be greatly amused as well as instructed by them, in many ways.

HORTICULTURAL ART JOURNAL is resplendent in Apples and Peaches, besides much valuable information for the Horticulturist.

STECHER LITHOGRAPHIC CO., Rochester, N. Y.

VICK'S ILLUSTRATED MONTHLY—is chiefly devoted to flora-culture, with much that is new and valuable upon that topic, with delicately colored illustrations of some new varieties of garden flowers. JAMES VICK, Rochester, N. Y.

OUR UNCLE AND AUNT.—The foregoing is the title of a handy volume of 223 pp. by Armala Martin, of Cairo, Ill., who has heretofore contributed to these columns. The author has presented in the form of an interesting narrative, the best argument in favor of Woman's Rights that has come under our notice. This, she has been able to do by thoroughly exposing Woman's *wrongs*, as heretofore and now sanctioned by law. She deals with the hackneyed term of "Woman's sphere," which every opposer of her claims to political equality with her male *master* is ready to cry out, with a keen sarcasm, and sustains her position by unanswerable argument. We heartily commend this volume to our readers of both sides of the question. Published by G. PUTNAM'S SONS, New York.

